# Effect of Training for an Athletic Challenge on Illness Cognition in Individuals with Chronic Disability: A Prospective Cohort Study

**DOI:** 10.3390/ijerph21010058

**Published:** 2023-12-30

**Authors:** Joy M. DeShazo, Ingrid Kouwijzer, Sonja de Groot, Marcel W. M. Post, Linda J. M. Valent, Christel M. C. van Leeuwen, Huacong Wen, Rachel E. Cowan, on behalf of the HandbikeBattle Group

**Affiliations:** 1 Department of Physical Medicine and Rehabilitation, University of Alabama at Birmingham, Birmingham, AL 35294, USA; joy.deshazo@encompasshealth.com (J.M.D.); hcwen@uab.edu (H.W.); recowan@uabmc.edu (R.E.C.); 2 Department of Human Movement Sciences, Faculty of Behavioural and Movement Sciences, Amsterdam Movement Sciences, Vrije Universiteit Amsterdam, 1081 BT Amsterdam, The Netherlands; s.de.groot@vu.nl; 3 Amsterdam Rehabilitation Research Center|Reade, 1054 HW Amsterdam, The Netherlands; 4 Center of Excellence for Rehabilitation Medicine, UMCU Brain Center, University Medical Center Utrecht and De Hoogstraat Rehabilitation, 3583 TM Utrecht, The Netherlands; m.post@dehoogstraat.nl (M.W.M.P.); c.v.leeuwen@dehoogstraat.nl (C.M.C.v.L.); 5 Center for Rehabilitation, University Medical Center Groningen, University of Groningen, 9713 GZ Groningen, The Netherlands; 6 Research and Development, Heliomare Rehabilitation Center, 1949 EC Wijk aan Zee, The Netherlands; l.valent@heliomare.nl

**Keywords:** spinal cord injury, adaptive sport, psychological adaptation, appraisals, longitudinal study, exercise, wheelchair

## Abstract

Illness cognitions (IC) influence how a patient adapts to a chronic disease. The aim was (1) to determine if training for a handcycling mountain time trial (HandbikeBattle) improves IC and (2) to identify factors associated with IC change scores. Persons with a chronic disability (N = 220; including N = 151 with spinal cord disorder) trained 5 months and participated in the time trial. The IC Questionnaire measured helplessness, acceptance, perceived benefits and was assessed before training (T1), after training (T2), and four months after the event (T3). Age, sex, body mass index (BMI), time since injury (TSI), disability characteristics, self-efficacy, mental health (MH) and musculoskeletal pain were obtained at T1. Multilevel regression analyses showed that helplessness decreased (from 11.96 to 11.28, *p* < 0.01) and perceived benefits increased (from 16.91 to 17.58, *p* < 0.01) from T1 to T2. For helplessness this decrease persisted during follow-up (11.16 at T3). Changes in helplessness were associated with self-efficacy (*p* = 0.02), MH (*p* = 0.02) and lesion completeness (*p* = 0.02), and were independent of disability type (*p* = 0.66), lesion level (*p* = 0.30) and demographics such as sex (*p* = 0.29) and age (*p* = 0.67). Training with peers may improve helplessness and perceived benefits in individuals with a chronic disability. Especially individuals with MH problems might benefit from training for an athletic challenge with peers to improve illness cognitions, and ultimately, quality of life.

## 1. Introduction

When individuals acquire a severe chronic disease, such as spinal cord injury (SCI), they are faced with life-long physical health consequences. In addition, life satisfaction is reduced and mental health problems are more common in individuals with SCI compared to the general population [1,2,3,4]. However, how individuals with SCI rate their quality of life is diverse and not necessarily related to the severity of the SCI [1,5]. It is assumed that the way individuals perceive their illness accounts for much of the individual differences in quality of life [6,7,8,9,10]. This is referred to as “illness cognitions”, “illness representations” or “appraisals”. These illness cognitions can be adaptive or maladaptive. Evers et al. [6] proposed three generic illness cognitions: “helplessness as a way of emphasizing the aversive meaning of the disease, acceptance as a way to diminish the aversive meaning, and perceived benefits as a way of adding a positive meaning to the disease.” Helplessness is a maladaptive illness cognition with negative impact on health-related behaviors and outcomes [6]. Acceptance and perceived benefits are positive adaptations with beneficial impact [6].

It is important to know more about illness cognitions and how to possibly influence illness cognitions as research in individuals with SCI showed strong negative associations between helplessness and well-being and strong positive associations between acceptance and well-being [11,12]. Two previous observational studies on change in illness cognitions in individuals with SCI showed inconclusive results. One study showed no changes in helplessness, acceptance and perceived benefits from inpatient rehabilitation to 6 months after discharge [13]. However, the other study showed a decrease of experienced illness threat associated with having SCI according to the illness representations at discharge compared to first inpatient rehabilitation admission [14]. In addition, older age and having a complete SCI were associated with higher threat at admission, but not at discharge [14].

Interventions targeting illness cognition have improved health behaviors and outcomes in diverse populations [15]. These interventions are often grounded in cognitive paradigms and have utilized motivational interviewing [16], mindfulness-based cognitive therapy [17], disease education [18], and education on illness perception [19]. Unfortunately, there is a lack of research into physical activity-types of interventions to improve illness cognition, whereas previous studies showed that such interventions are associated with improved self-efficacy [20], increased acceptance of disability [21], improved physical and mental empowerment [22], a greater sense of regaining control over the body [22], improved body satisfaction [23] and life satisfaction [24].

In addition, few studies have examined the associations between demographic and health-related factors and responsiveness to an illness cognition intervention. From previous, primarily cross-sectional, studies it is known that poor mental health and pain are consistently associated with maladaptive illness cognition in multiple populations [12,25,26,27,28,29]. In contrast, factors such as age [6,30,31,32], sex [6,32], illness duration [6,30], and Body Mass Index (BMI) [33,34] have shown inconsistent associations with illness cognition.

The purpose of this study is to assess if participation in training for a challenging handcycling event improves illness cognition in persons with a physical disability. Our hypothesis is that training for a handcycling mountain time trial (the HandbikeBattle) improves illness cognition. Our aims are twofold; (i) to describe changes in illness cognition during training and after participation, and (ii) to identify which participant-centered factors are associated with changes in illness cognition.

## 2. Materials and Methods

This study utilized data collected in the ongoing HandbikeBattle project.

### 2.1. The HandbikeBattle

The HandbikeBattle event is an annual 20.2 km mountain time trial each June. It was founded in 2013 by participating rehabilitation centers with the purpose of encouraging persons with chronic disabilities to participate in regular physical activity. Twelve Dutch rehabilitation centers participate in the event each year. Each center competes with a team consisting of former rehabilitation patients with a chronic disability, for example SCI or amputation. The 5-month training period is free-living, that is, no specific training program is provided by the researchers. Connected to the HandbikeBattle is an observational cohort study that was initiated to monitor effects of participation in the training period and the event. All HandbikeBattle participants were asked to participate in the study. They enrolled voluntarily. The study was approved by the local ethics committee of the Center for Human Movement Sciences, University Medical Center Groningen, The Netherlands (ECB/2012_12.04_l_rev/Ml). Each participant signed an informed consent before the first assessment.

### 2.2. Participants

Participants in the current study were former patients of twelve rehabilitation centers who participated in the HandbikeBattle between 2013 to 2018. A physician medically screened each participant prior to the start of training. Exclusion criteria included any contraindication for exercise according to the American College of Sports Medicine guidelines [35]. In addition, sufficient knowledge of the Dutch language was a prerequisite to understand the instructions and questionnaires. Participants were included in the current study if they filled out the questionnaires during at least two out of three time points.

### 2.3. Data Collection

**Figure 1:**
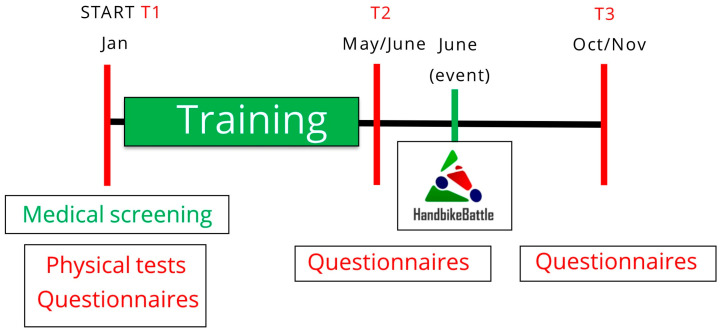
Study design. Measurements are performed at the start of the training period (January, T1); after the training period, prior to the event (June, T2); and follow-up, four months after the event (October/November, T3).

Testing occurred at each participating center’s designated test sites in January (T1) and June (T2) (Figure 1). These months, respectively, correspond to pre-training and post-training (just before the event). At T1, demographic information (age and sex), disability characteristics, physical fitness and BMI were collected. Psychological questionnaires were administered at T1, T2 and October/November (T3), i.e., post-participation. The questionnaires were part of a set of questionnaires which took participants 30–45 min to complete in total. Participants were invited by e-mail with a link and could fill out the questionnaires in their own time at home.

### 2.4. Questionnaires

#### 2.4.1. Illness Cognition Questionnaire

Illness cognition was quantified at T1, T2, and T3 with the self-reported Illness Cognition Questionnaire (ICQ), which consists of 18 items that are scored on a 4-point scale (1 = not at all, 2 = somewhat, 3 = to a large extent, 4 = completely) (Supplementary Table S1). The ICQ has three sub-scales; helplessness (6 items), acceptance (6 items), and perceived benefits (6 items) [6]. Helplessness includes cognitions emphasizing the aversive meaning of the illness (e.g., “My illness limits me in everything that is important to me”), acceptance includes cognitions diminishing the aversive meaning of the illness (e.g., “I have learned to accept the limitations imposed by my illness”), and perceived benefits includes cognitions giving a positive meaning to the illness (e.g., “Dealing with my illness has made me a stronger person”) [6]. Each subscale ranges from 6 to 24 and is computed by summing responses to each sub-scale item. Helplessness is computed by summing questions 1, 5, 7, 9, 12 and 15. Acceptance is computed by summing questions 2, 3, 10, 13, 14 and 17. Perceived benefits is computed by summing questions 4, 6, 8, 11, 16 and 18. Higher scores correspond to greater levels of the construct. ICQ validity and reliability have been demonstrated in individuals with multiple sclerosis and rheumatoid arthritis [6]. Previous studies have administered the ICQ to individuals with SCI [11,12,13,36].

#### 2.4.2. Mental Health Inventory

The self-reported 5-item Mental Health Inventory (MHI-5) of the SF-36 quantified mental health at T1. It requires respondents to indicate the frequency of nervousness, sadness, peacefulness, depressed mood, and happiness during the previous 4 weeks using a 6-point Likert scale (1 = all the time to 6 = none of the time). A total score between 0 (lowest mental health) and 100 (highest mental health) was computed. MHI-5 scores at or below 72 indicate mental health problems are present [37]. For analyses, participants were dichotomized as having (MHI-5 ≤ 72) or not having (MHI-5 > 72) mental health problems. The MHI has been shown to be reliable and valid in individuals with SCI [38].

#### 2.4.3. Self-Efficacy

Self-efficacy was measured with the self-reported general self-efficacy scale [39,40] at T1. It consists of 16 items graded on a 5-point Likert scale ranging from strongly disagree to strongly agree. A total score between 16 (low self-efficacy) and 80 (high self-efficacy) was computed. It has good re-test reliability and internal consistency [39,40] and has been used in other SCI studies [11,13].

#### 2.4.4. Pain Assessment

Participants were asked in a standardized self-reported questionnaire whether they experienced pain in the joints or muscles of both upper extremities at T1. If pain was present, participants were asked to indicate the location and rate the severity (1 = no pain, 2 = very mild, 3 = mild, 4 = moderate, 5 = severe, and 6 = very severe). For analyses participants were dichotomized into no/mild pain (rated 0–3) or moderate/severe pain (rated ≥ 4) [41].

#### 2.4.5. Sports Participation

The hours of sports participation per week were self-reported by the participants at T2 and T3.

### 2.5. Physical Determinants

#### 2.5.1. Disability Etiology

Participants with SCI and spina bifida were grouped together for analysis. Participants with other injury types (e.g., amputation and multi trauma) were categorized as “other”.

#### 2.5.2. SCI Lesion Characteristics

SCI was characterized according to the 2011 International Standards for Neurological Classification of Spinal Cord Injury [42]. Paraplegia was defined as neurologic levels at or below T2 and tetraplegia was defined as neurologic levels at or above T1. Motor complete corresponded to AIS grades A and B and motor incomplete corresponded to AIS grades C and D.

#### 2.5.3. Time Since Injury

For all participants, time since injury (TSI) was determined as the time between occurrence of injury and the first measurement time (T1).

#### 2.5.4. Physical Fitness

Physical fitness was defined as peak power output (POpeak, watts (W)) determined during a synchronous arm crank aerobic exercise test to volitional exhaustion. Either a 1-min step protocol or continuous ramp protocol was used and was individualized for each participant. For the 1-min protocol, POpeak was defined as the highest PO that was maintained for at least 30 seconds. For the ramp protocol, the highest PO achieved during the test was considered POpeak.

#### 2.5.5. Body Mass Index

BMI was calculated for each participant with the following formula: body mass (kg)/height (m)^2^. Body mass was measured with a wheelchair scale and height was recalled by the participants.

### 2.6. Statistical Analysis

The analyses were performed with SPSS (Version 28.0; IBM Corp., Armonk, NY, USA) and MLwiN version 2.36 [43]. Demographic and health-related factors of the study participants were analyzed by using descriptive statistics, including mean and percentage. Data were tested for normality with the Kolmogorov-Smirnov test with Lilliefors significance correction, combined with z scores for skewness and kurtosis. To ascertain possible response bias, characteristics of included participants in the present study were compared with non-participants (i.e., drop-outs or those who did not fill out all questionnaires) using *t*-tests, Mann-Whitney U tests, and chi-square tests.

Multilevel analyses were conducted to be able to make adjustments for the dependency of the observations within participants and participants within centers. An additional advantage of multilevel analyses is the robustness for missing data [44].

To test the first aim, multilevel models with three levels were created; with observations within participants as first level, participant as second level, and rehabilitation center as third level. Three models were created to examine the longitudinal trajectory of illness cognition: one with helplessness, one with acceptance and one with perceived benefits as outcome measure. Time (T1, T2, T3) was included as a categorical variable with two dummies.

To test the second aim, exploratory analyses were performed in which interaction terms with the time dummies were investigated in a series of separate models for each of the following T1 determinants: age (years), sex (male = 1, female = 2), BMI (kg/m^2^), disability etiology (SCI/SB = 0, other = 1), TSI (years), lesion completeness (motor complete = 0, motor incomplete = 1), level of injury (tetraplegia = 0, paraplegia = 1), POpeak (W), musculoskeletal pain (no-mild = 0, moderate-severe = 1), self-efficacy, mental health (mental health problems MHI-5 ≤ 72 = 0, no mental health problems MHI-5 > 72 = 1).

## 3. Results

In total, 319 individuals with chronic physical disabilities started training. Forty-three individuals dropped out due to medical reasons (N = 20), motivational problems (N = 12), being too busy with work/family (N = 5) or personal/unknown reasons (N = 6). Fifty-six individuals did not fill out questionnaires at any time-point (N = 25), or only at one time-point (N = 31). Hence, 99 out of 319 individuals were not included in the present study and defined as non-participants. This resulted in 220 participants to be included. The sample for this study included 151 individuals with SCI or spina bifida, and 69 with other disabilities. Participants were on average older and had a higher POpeak than non-participants (Table 1). Descriptive data of the IC outcome measures at all time-points are depicted in Table 2. The mean hours of sports participation at T2 and T3 was 8.2 ± 3.2 h/week and 5.8 ± 4.0 h/week, respectively.

### 3.1. Longitudinal Changes in Illness Cognition

Helplessness showed a significant improvement between T1 (start of training) and T2 (after training), whereas no significant change was found during follow-up (T3) (Table 3). When the model was recalculated with T1 as reference category, there was a significant improvement between T1 and T3 (Table 3). Acceptance showed no significant changes over time (Table 3). Perceived benefits showed a significant improvement between T1 and T2, whereas no significant change was found during follow-up (T3) (Table 3). When the model was recalculated with T1 as reference category, there was no significant change between T1 and T3 (Table 3).

### 3.2. Factors Associated with Change in Illness Cognition

**Figure 2:**
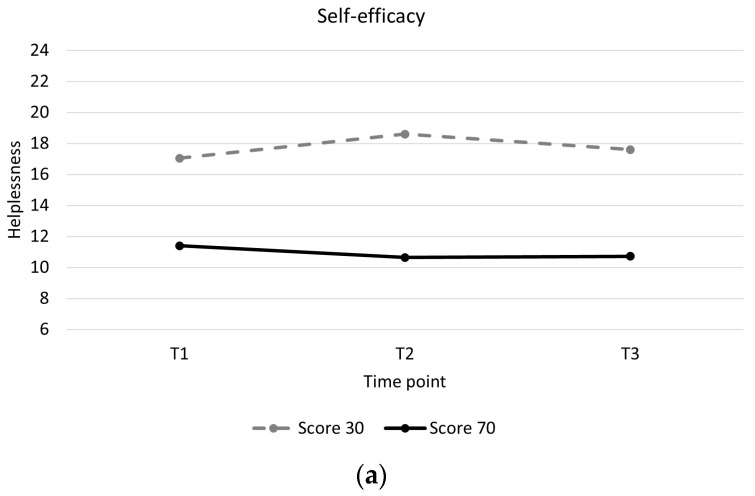

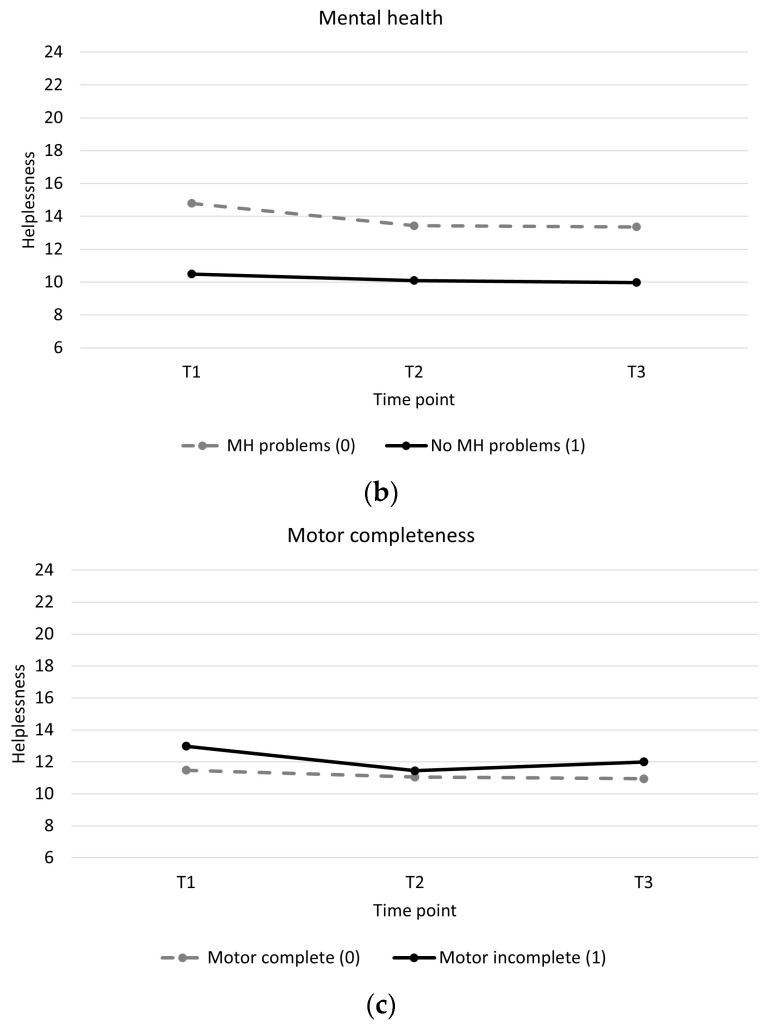
Longitudinal trajectory of helplessness with interaction effects. (**a**): Helplessness for a participant with a self-efficacy score of 30 (low self-efficacy) versus a participant with a self-efficacy score of 70 (high self-efficacy). (**b**): Helplessness for participants with mental health problems (0) versus participants without mental health problems (1). (**c**): Helplessness for participants with a motor complete spinal cord injury (0) versus participants with a motor incomplete spinal cord injury (1).

Individuals with low baseline self-efficacy showed higher levels of helplessness throughout and showed a slight increase in helplessness during training compared to individuals with high baseline self-efficacy, who showed a slight decrease in helplessness (Table 4, Figure 2a). Individuals with mental health problems showed higher levels of helplessness throughout and greater decreases in helplessness between pre and post training than individuals without mental health problems (Table 4, Figure 2b). Individuals with a motor incomplete injury showed greater decreases in helplessness than individuals with motor complete injury (Table 4, Figure 2c). There were no significant associations between changes in acceptance and perceived benefits over time and baseline participant characteristics (Supplementary Material Tables S2 and S3).

## 4. Discussion

We investigated the effect of training for a handcycling mountain time trial on illness cognition in individuals with chronic disability. It appears that participation in training for the HandbikeBattle can improve illness cognition specifically by decreasing helplessness and increasing perceived benefits. For helplessness, this effect appears to persist for several months after participation in the time trial. Self-efficacy, mental health and lesion completeness at baseline were associated with change in helplessness during the training period, whereas, among others, disability etiology (SCI/SB versus other) was not.

Participants in the current study showed helplessness scores within the same range found in previous studies with patients with SCI, stroke, multiple sclerosis and rheumatoid arthritis (median and mean ranging from 11.3 to 13.4) [6,13,45]. The acceptance scores were within the same range as scores in previous studies as well (median and mean ranging from 15.8 to 19.0) [6,13,45]. The pre-training perceived benefits scores were higher than in previous studies (median and mean ranging from 15.0 to 15.5) [6,13,45]. Possible differences might be due to TSI. The participants in the current study had an average TSI of 12 years and were in the chronic phase of their disease, whereas some of the previous studies included patients within or shortly after inpatient rehabilitation.

We observed small but significant decreases in helplessness and small but significant increases in perceived benefits after five months of free-living handbike training and these improvements were sustained four months after participation in the HandbikeBattle. The improvement in helplessness was 4.4% for the total group from pre-training (T1) to follow-up (T3), whereas this improvement was 8.0% for the subgroup with mental health problems. Although improvements are small, previous studies on illness cognition interventions showed similar results. In the study by Callahan et al. [46] effects of a 6-week walking program for adults with arthritis were evaluated. They found a significant improvement in helplessness of 5.5% with an effect size of 0.24–0.28. Sararoudi et al. [18] studied an illness perception intervention given by a mental health counselor in patients with myocardial infarction. They reported an improvement of 7.1% in illness perception, 3 months after discharge.

The effects that were found in the present study may be explained by the role of exercise in alleviating stress and pain, and improving resiliency, personal control, self-efficacy, social integration and functional independence [47,48,49,50,51,52,53]. Anecdotal evidence suggests that social integration and peer support play an important role during training for and participating in the HandbikeBattle event. Unfortunately, we did not collect data with the participants in the current study to support this.

It is interesting that the decrease in helplessness was sustained several months after the event despite evidence that the stress-relieving benefits of exercise significantly decline after two weeks of exercise cessation [47]. Based on the self-reported hours of sports participation, it seems that most participants still participate in sports after the HandbikeBattle, but spend less hours on sports participation than during the training period. More detailed information about participants’ exercise habits following event completion would help clarify whether maintenance of decreased helplessness is mediated by continued exercise.

Our findings regarding associations between pre-training participant-centered factors and change in illness cognition are both supported and contradicted by the literature. Cross-sectional associations between illness cognition and mental health [12,45,54,55] and self-efficacy [56,57,58] are consistently reported in the existing literature. The results in the present study are in line with these findings. However, it is unclear whether the association with motor incompleteness is supported by literature. Kuiper et al. [14] showed that motor complete SCI was associated with higher threat at admission. At discharge there was no association with motor completeness. The findings of Kuiper et al. [14] could suggest that those with motor complete SCI experience a stronger decrease of threat compared to those with motor incomplete SCI. Though one would indeed expect individuals with less severe SCI to have lower levels of helplessness than those with more severe injury, the findings in the current study supports research that demonstrates poorer psychological outcomes in individuals with motor incomplete injuries [59]. Previously cited reasons for this finding include shorter acute rehabilitation stays, frustration with slowness of ambulation, anxiety about reinjury, and feeling misunderstood due to having less visible deficits [59]. More research is needed to confirm the role of lesion completeness in illness cognition.

### 4.1. Practical Implications

Overall we can conclude that in individuals with a chronic disability, small improvements in illness cognition can be achieved with training for a challenging handcycling event with peers. Therefore, physical activity-types of interventions might help to improve illness cognition, alongside cognitive interventions. In addition, we showed that changes in illness cognitions are more or less independent of disability type (SCI/SB versus other), lesion level and demographics such as sex and age. It might be that the way individuals perceive their illness is not necessarily dependent on the type and severity of the disease, but on other (psychological) factors. We showed that especially individuals with mental health problems showed a decrease in helplessness. Therefore, especially this group might benefit from training for and participating in an athletic challenge with peers to improve illness cognitions, and ultimately, quality of life. These findings concur with previous longitudinal HandbikeBattle studies that showed improvements in body satisfaction and life satisfaction during training [23,24]. A change in illness cognition might be another piece in the multifactorial puzzle of improvements in quality of life during training and physical activity.

### 4.2. Study Limitations

There might be a selection bias, as individuals who dropped out before or during the training period were not included in the analyses. Therefore, individuals with a very low cardiorespiratory fitness might be less prominent in the present study. In addition, individuals who are well-adjusted to their disability may be more likely to sign up for the HandbikeBattle. It is, however, important to mention that the event was created to push physical and mental boundaries for individuals who might need this and that participants were not professional athletes. Second, we only measured musculoskeletal pain and no other causes of pain such as neuropathic pain. Therefore, we are unsure whether pain in general might effect changes in illness cognition. Third, we are unable to comment on what social aspects of the training, such as social integration and peer support, might have contributed to the observed changes. Last, there is a potential risk of bias with self-reported questionnaires. For example, it has been described that individuals might overestimate their level of physical activity and sports participation [60]. In that respect objective measures are more appropriate to measure physical activity. In future studies, qualitative methods might aid in unraveling more details on the change in illness cognition, associated factors and whether individuals perceive this change as meaningful.

## 5. Conclusions

Training with peers may improve helplessness and perceived benefits in individuals with a chronic disability. For helplessness, this change appears to persist for several months during follow-up. Changes in illness cognitions are more or less independent of disability type, lesion level and demographics such as sex and age. Especially mental health and self-efficacy are important determinants for (change in) helplessness.

## Figures and Tables

**Table 1:** Demographic characteristics, injury-related characteristics, and baseline assessments of participants and non-participants.

Characteristics	N	Participants		N	Non-Participants	
Sex (male/female) (%male)	220	164/56	(75%)	99	75/24	(76%)
Age (years)	220	41 ± 13 *		97	36 ± 12 *	
Disability etiology	220			91		
Spinal cord injury/Spina bifida		151	(69%)		64	(70%)
Other		69	(31%)		27	(30%)
Time since injury (years)	199	12 ± 13		79	13 ± 13	
<2 years		39	(19%)		11	(14%)
2–9 years		71	(36%)		28	(35%)
>9 years		89	(45%)		40	(51%)
Body Mass Index (kg/m^2^)	210	24.6 ± 4.5		77	24.9 ± 5.6	
POpeak (W)	189	117 ± 37 *		70	104 ± 34 *	
Self-efficacy	121	68.8 ± 9.0		20	63.3 ± 12.8	
Illness Cognition						
Helplessness	200	12.0 ± 3.9		38	11.1 ± 4.2	
Acceptance	199	18.7 ± 4.1		38	18.3 ± 4.7	
Perceived benefits	199	17.1 ± 4.8		38	17.9 ± 4.4	
Mental Health Inventory	200	76.5 ± 15.4		39	72.1 ± 18.2	
≤72 (mental health problems)		68	(34%)		19	(49%)
>72 (no mental health problems)		132	(66%)		20	(51%)
Musculoskeletal pain (no-mild/moderate-severe) (%no-mild)		120/79	(60%)		23/18	(56%)
Completeness of injury (SCI only)	131			53		
Motor complete		86	(66%)		33	(62%)
Motor incomplete		45	(34%)		20	(38%)
Level of injury (SCI only)	135			57		
Tetraplegia		21	(16%)		11	(19%)
Paraplegia		114	(84%)		46	(81%)

Data represent N (%) or mean ± SD. POpeak: peak power output; Musculoskeletal pain: two categories: (1) no-mild pain and (2) moderate-severe pain.* Significant difference with *p* < 0.05 between participants and non-participants.

**Table 2:** Illness cognition of participants at all time points.

	T1			T2			T3		
	N	Mean ± SD	Median [IQR]	N	Mean ± SD	Median [IQR]	N	Mean ± SD	Median [IQR]
Helplessness (6–24)	200	12.0 ± 3.9	11.0 [9.0–14.8]	207	11.3 ± 3.8	11.0 [9.0–13.0]	179	11.1 ± 3.8	10.0 [8.0–13.0]
Acceptance (6–24)	199	18.7 ± 4.1	19.0 [16.0–22.0]	206	18.8 ± 3.9	19.0 [16.0–22.0]	179	18.8 ± 3.9	19.0 [16.0–22.0]
Perceived benefits (6–24)	199	17.1 ± 4.8	17.0 [14.0–21.0]	206	17.6 ± 4.8	18.0 [14.0–22.0]	178	17.2 ± 4.7	17.0 [14.0–21.3]

Data represent N (%), mean (SD) and median [IQR]. T1 = start of the training period. T2 = after the training period, prior to the HandbikeBattle event. T3 = follow-up measurement, four months after the event.

**Table 3:** Longitudinal trajectory of illness cognition.

	Helplessness		Acceptance		Perceived Benefits	
	Beta (SE)	*p*-Value	Beta (SE)	*p*-Value	Beta (SE)	*p*-Value
Constant (reference T2)	11.277 (0.262)		18.806 (0.270)		17.577 (0.324)	
∆ T2–T1	0.678 (0.187)	<0.01 *	−0.245 (0.201)	0.22	−0.669 (0.219)	<0.01 *
∆ T2–T3	−0.121 (0.195)	0.53	−0.086 (0.209)	0.68	−0.338 (0.229)	0.14
Constant (reference T1)	11.956 (0.263)		18.560 (0.271)		16.908 (0.326)	
∆ T1–T2	−0.678 (0.187)	<0.01 *	0.245 (0.201)	0.22	0.669 (0.219)	<0.01 *
∆ T1–T3	−0.800 (0.198)	<0.01 *	0.160 (0.212)	0.45	0.331 (0.232)	0.15

T1 = start of the training period. T2 = after the training period, prior to the HandbikeBattle event. T3 = follow-up measurement, four months after the event. * Significance with *p* < 0.05. The ∆ T2–T1 = a negative regression coefficient representing an increase of the dependent variable over time. The ∆ T2–T3 = a negative regression coefficient representing a decrease of the dependent variable over time.

**Table 4:** Longitudinal trajectory of helplessness with interaction effects.

Determinants	Constant (Reference:T2)	∆ T2–T1	∆ T2–T3	Determinant	(∆ T2–T1) × Determinant	(∆ T2–T3) × Determinant
Sex (reference: male)	11.411 (0.302)	0.565 (0.215) *	−0.145 (0.227)	−0.536 (0.602)	0.455 (0.432)	0.120 (0.440)
Age, years	9.720 (0.864)	0.923 (0.628)	−0.386 (0.653)	0.038 (0.020)	−0.006 (0.014)	0.006 (0.015)
Disability etiology (reference: SCI/SB)	11.189 (0.315)	0.629 (0.222) *	0.054 (0.239)	0.283 (0.563)	0.180 (0.408)	−0.503 (0.412)
Time since injury (years)	11.503 (0.365)	1.030 (0.270) *	−0.131 (0.275)	−0.026 (0.021)	−0.025 (0.015)	0.003 (0.015)
Body Mass Index, kg/m^2^	8.165 (1.449)	−0.215 (1.044)	0.301 (1.154)	0.126 (0.058) *	0.036 (0.042)	−0.016 (0.046)
POpeak (W)	11.630 (0.943)	0.796 (0.692)	0.764 (0.707)	−0.002 (0.008)	−0.001 (0.006)	−0.008 (0.006)
Self-efficacy	24.569 (2.573)	−3.299 (1.769)	−1.811 (1.929)	−0.199 (0.037) *	0.058 (0.025) *	0.027 (0.028)
Mental Health (reference: ≤72 (mental health problems))	13.439 (0.417)	1.355 (0.322) *	0.192 (0.361)	−3.337 (0.514) *	−0.960 (0.398) *	−0.315 (0.440)
Musculoskeletal pain (reference: no-mild pain)	10.836 (0.352)	0.669 (0.247) *	0.117 (0.272)	1.074 (0.558)	0.143 (0.392)	−0.341 (0.425)
Lesion completeness (reference: motor complete)	11.047 (0.385)	0.435 (0.263)	−0.097 (0.289)	0.401 (0.659)	1.101 (0.460) *	0.652 (0.478)
Level of injury (reference: tetraplegia)	10.283 (0.786)	1.320 (0.556) *	0.461 (0.568)	1.138 (0.855)	−0.629 (0.601)	−0.395 (0.619)

Data represent beta (SE). Eleven separate models were created (one model for each determinant). Each model consisted of the time dummies, one determinant, and the interaction effect between time and determinant. Sex: male = 1, female = 2, reference: male. Disability etiology: two categories: (1) SCI/SB = 0 and (2) other = 1, reference: SCI/SB. POpeak: peak power output; Mental health: two categories: (1) mental health problems = 0 and (2) no mental health problems = 1, reference: mental health problems. Musculoskeletal pain: two categories: (1) no-mild pain = 0 and (2) moderate-severe pain = 1, reference: no-mild pain. Lesion completeness: two categories: (1) motor complete = 0 and (2) motor incomplete = 1, reference: motor complete. Level of injury: two categories: (1) tetraplegia = 0 and (2) paraplegia = 1, reference: tetraplegia. T1 = start of the training period. T2 = after the training period, prior to the HandbikeBattle event. T3 = follow-up measurement, 4 months after the event. * Significance with *p* < 0.05.

## Data Availability

The datasets generated during and/or analyzed during the current study are available from the corresponding author on reasonable request.

## Supplementary Materials

The following supporting information can be downloaded at: https://www.mdpi.com/article/10.3390/ijerph21010058/s1, Table S1: Illness Cognition Questionnaire; Table S2: Longitudinal trajectory of acceptance with interaction effects; Table S3: Longitudinal trajectory of perceived benefits with interaction effects.
