# Supplementary material for: Effect of Training for an Athletic Challenge on Illness Cognition in Individuals with Chronic Disability: A Prospective Cohort Study
Source: Int J Environ Res Public Health. 2023 Dec 30;21(1):58. doi: 10.3390/ijerph21010058 (PMC10815898; doi:10.3390/ijerph21010058)
Supplement: Supplementary file 1 [file ijerph-21-00058-s001.zip › Table S1.pdf]

Table S1. Illness Cognition Questionnaire

		Not at all	Somewhat	To a large extent	Completely
1.	Because of my illness, I miss the things I like to do most.	1	2	3	4
2.	I can handle the problems related to my illness.	1	2	3	4
3.	I have learned to live with my illness.	1	2	3	4
4.	Dealing with my illness has made me a stronger person.	1	2	3	4
5.	My illness controls my life.	1	2	3	4
6.	I have learned a great deal from my illness.	1	2	3	4
7.	My illness makes me feel useless at times.	1	2	3	4
8.	My illness has made life more precious to me.	1	2	3	4
9.	My illness prevents me from doing what I would really like to do.	1	2	3	4
10.	I have learned to accept the limitations imposed by my illness.	1	2	3	4
11.	Looking back, I can see that my illness has also brought about some positive changes in my life.	1	2	3	4
12.	My illness limits me in everything that is important to me.	1	2	3	4
13.	I can accept my illness well	1	2	3	4
14.	I think I can handle the problems related to my illness, even if the illness gets worse.	1	2	3	4
15.	My illness frequently makes me feel helpless.	1	2	3	4
16.	My illness has helped me realize what's important in life.	1	2	3	4
17.	I can cope effectively with my illness.	1	2	3	4
18.	My illness has taught me to enjoy the moment more.	1	2	3	4
