# Supplementary material for: Effect of Training for an Athletic Challenge on Illness Cognition in Individuals with Chronic Disability: A Prospective Cohort Study
Source: Int J Environ Res Public Health. 2023 Dec 30;21(1):58. doi: 10.3390/ijerph21010058 (PMC10815898; doi:10.3390/ijerph21010058)
Supplement: Supplementary file 1 [file ijerph-21-00058-s001.zip › Table S3.pdf]

Table S3. Longitudinal trajectory of perceived benefits with interaction effects.

Determinants	Constant (reference:T2)	Δ T2 – T1	Δ T2 – T3	Determinant	(Δ T2 – T1) x determinant	(Δ T2 – T3) x determinant
Sex (reference: male)	17.711 (0.372)	-0.581 (0.252)*	-0.224 (0.266)	-0.525 (0.747)	-0.359 (0.509)	-0.432 (0.519)
Age, years	21.0441 (1.059)	-0.156 (0.745)	-0.679 (0.776)	-0.083 (0.024)*	-0.012 (0.017)	0.008 (0.018)
Disability etiology (reference: SCI/SB)	17.770 (0.390)	-0.604 (0.262)*	-0.340 (0.281)	-0.612 (0.696)	-0.223 (0.479)	0.005 (0.484)
Time since injury (years)	17.534 (0.460)	-0.846 (0.316)*	-0.495 (0.321)	0.002 (0.027)	0.009 (0.018)	0.012 (0.018)
Body Mass Index, kg/m^2	18.071 (1.830)	-0.590 (1.234)	-0.812 (1.369)	-0.018 (0.073)	-0.002 (0.049)	0.019 (0.055)
POpeak (W)	15.695 (1.138)	-0.563 (0.793)	0.029 (0.811)	0.016 (0.009)	-0.001 (0.007)	-0.004 (0.007)
Self-efficacy	10.273 (3.295)	-0.834 (2.190)	2.570 (2.388)	0.112 (0.047)*	0.004 (0.031)	-0.036 (0.034)
Mental Health (reference: ≤ 72 (mental health problems))	16.797 (0.575)	-0.638 (0.368)	-0.552 (0.414)	1.430 (0.710)*	-0.032 (0.456)	0.428 (0.505)
Musculoskeletal pain (reference: no-mild pain)	17.819 (0.440)	-0.600 (0.281)*	-0.005 (0.310)	-0.283 (0.696)	-0.153 (0.445)	-0.607 (0.484)
Lesion completeness (reference: motor complete)	18.054 (0.516)	-0.518 (0.272)	-0.280 (0.300)	-0.761 (0.883)	-0.675 (0.477)	-0.113 (0.496)
Level of injury (reference: tetraplegia)	18.548 (1.048)	-1.384 (0.578)*	-0.243 (0.591)	-0.942 (1.140)	0.751 (0.626)	-0.103 (0.644)

Data represent beta (SE). Eleven separate models were created (one model for each determinant). Each model consisted of the time dummies, one determinant, and the interaction effect between time and determinant. Sex: male = 1, female = 2, reference: male. Disability etiology: two categories: (1) SCI/SB = 0 and (2) other = 1, reference: SCI/SB. POpeak: peak power output; Mental health: two categories: (1) mental health problems = 0 and (2) no mental health problems = 1, reference: mental health problems. Musculoskeletal pain: two categories: (1) no-mild pain = 0 and (2) moderate-severe pain = 1, reference: no-mild pain. Lesion completeness: two categories: (1) motor complete = 0 and (2) motor incomplete = 1, reference: motor complete. Level of injury: two categories: (1) tetraplegia = 0 and (2) paraplegia = 1, reference: tetraplegia. T1 = start of the training period. T2 = after the training period, prior to the HandbikeBattle event. T3 = follow-up measurement, 4 months after the event. * Significance with $p < 0.05$.
